# Psychometric evaluation of the Chinese version of the Academic Resilience Scale-30 (C-ARS-30) in college students

**DOI:** 10.3389/fpsyg.2024.1276618

**Published:** 2024-08-07

**Authors:** Wen-ying Tan, Jia-ni Chen, Sui-hua Lu, Chun-qin Liu, Qing Luo, Yu Ma, Ying Zhou, Thomas K. S. Wong, Hui-fang Chen, Li-qin Song, Chu-yuan Miao, Jing-wen Chen, Graeme D. Smith

**Affiliations:** ^1^School of Nursing, Guangzhou Medical University, Guangzhou, Guangdong, China; ^2^School of Guangzhou Health Science College, Guangzhou, Guangdong, China; ^3^School of Health Sciences, Caritas Institute of Higher Education, Tseung Kwan O, Hong Kong SAR, China

**Keywords:** resilience, academic resilience, college student, reliability, validity

## Abstract

**Background:**

Amidst the expansion of student enrollment in higher education, the well-being and retention rates of students have emerged as important concerns. Resilience, especially academic resilience, a multidimensional construct that can lead to academic success in adversity, is pivotal in enabling students to successfully cope with academic challenges. While the Academic Resilience Scale-30 (ARS-30) has been validated as an effective instrument in various languages, its applicability for Chinese students in higher education remains unexplored.

**Objective:**

This study aims to translate and validate the ARS-30 in Chinese, assessing its reliability and validity among Chinese college students in higher education.

**Methods:**

A convenience sample of 1,542 students participated in this study. The inventory included the demographic form, Chinese version of ARS-30 (C-ARS-30), 10-item Connor Davidson Resilience Scale (CD-RISC-10), and General Self-Efficacy Scale (GSES). The assessment of validity was conducted by analyzing content validity, construct validity, convergent and discriminant validity, as well as criterion-related validity. Construct validity was evaluated through Confirmatory Factor Analysis (CFA), Exploratory Factor Analysis (EFA) and Exploratory Structural Equation Modeling (ESEM). Reliability analysis was performed using Cronbach’s alpha and test–retest reliability.

**Results:**

The C-ARS-30 demonstrated commendable content validity, with the CVI value of items ranging from 0.833 to 1.000, and a total scale CVI of 0.986. ESEM analysis revealed a solid four-factor structure, maintaining the scale’s 30 items with excellent fit indices (χ^2^/df = 2.647, CFI = 0.937, TLI = 0.915, RMSEA = 0.057, SRMR = 0.027). The total score of C-ARS-30 exhibited positive correlations with the CD-RISC-10 (*r* = 0.542) and the GSES (*r* = 0.488). The scale demonstrated high internal consistency (Cronbach’s *α* = 0.930) and test–retest reliability (0.794, *p* < 0.01).

**Conclusion:**

The C-ARS-30 is a reliable and valid instrument for assessing academic resilience among Chinese college students, offering a valuable tool for educational and psychological evaluations.

## 1 Introduction

In the context of higher education in China, which includes universities and both technical and vocational colleges, students commonly encounter various stressors throughout their academic journey. Significant stress arises from academic challenges such as peer competition, grade pressure, and exam stress, personal obstacles like concerns over education quality and social interaction deficiencies, and negative life events including exam failure and public embarrassment (Li et al., 2005; Liu et al., 2023). Stressors related to the academic perspective appear to dominate, highlighting that academic concerns are the primary focus (Li et al., 2005). Furthermore, prolonged psychological stress is strongly linked to mental health disorders (Zhang et al., 2022). Students experiencing higher level of stress are more susceptible to acute stress disorder symptoms and exhibit reduced resilience (Ye et al., 2020). This stress can negatively impact students’ academic performance (Bruffaerts et al., 2018), and even increases university dropout rates (Eisenberg et al., 2009).

Resilience is defined as the dynamic system’s ability to adapt to disruptions threatening its survival, function, or development (Masten, 2014). As an individual protective factor, resilience is reflected in how a person copes with difficulties and responds to future challenges (Mawdsley and Willis, 2023). Within the educational field, academic resilience, which is closely related to resilience, has been proposed as a crucial psychological factor that deal with challenges and fosters academic success (Martin and Marsh, 2009). Academic resilience, as with resilience, has been defined from a variety of perspectives (Mancini and Bonanno, 2009; Martin, 2013; Stainton et al., 2019; Liu et al., 2020). From an ability perspective, it refers to an individual’s capacity to overcome acute or chronic academic adversity that pose a significant threat to the educational process, making adaptive adjustments and achieving academic success (Martin and Marsh, 2009). The study of academic resilience in the higher educational setting provides valuable information on ways to help enhance academic success and reduce attrition rates in undergraduate students (Cassidy et al., 2023).

Whilst, resilience is often associated with traumatic situations, academic resilience specifically addresses the stressors and adversities encountered in educational settings, which, although not always traumatic, significantly impact students’ academic success and well-being. Academic resilience is vital for the emotional well-being of students in the higher education (Hwang and Shin, 2018). Studies indicate that academic resilience inversely relates to academic anxiety and stress, while positively correlating with academic achievement (Ragusa et al., 2023). Higher levels of academic resilience in students are linked to better coping skills in academic settings and lower incidences of burnout (Fiorilli et al., 2020; Tran et al., 2023).

Academic resilience is a crucial factor that helps students cope with stress and significant challenges. Valid and reliable tools for assessing academic resilience are vital for both understanding its importance and facilitating research in this domain. Key assessment tools used among college students include:

Academic Resilience Scale (ARS) by Martin and Marsh (2006): this early scale is unidimensional and was initially designed for primary and secondary school students. It comprises six items that assist students in evaluating their capacity to manage challenges and stress within the educational environment. The total scale demonstrates acceptable internal consistency, with a Cronbach’s α coefficient of 0.89. The ARS is widely utilized in educational settings for timely assessment of academic resilience.

Academic Resilience Scale-30 (ARS-30) by Cassidy (2016): uniquely focused on context-based measurement, the ARS-30 effectively captures the essence of academic resilience under stress. It presents students with a hypothetical scenario of academic failure and assesses their adaptive and maladaptive cognitive, emotional, and behavioral responses to such challenges. This tool includes three dimensions with 30 items, demonstrating strong reliability (Cronbach’s *α* coefficient of 0.90, and dimension-specific coefficients ranging from 0.78 to 0.83).

In addition to the two widely used assessment tools mentioned above, there has been recent attention on some specific scales. The Pharmacy Academic Resilience Scale by Chisholm-Burns et al. (2019) is adapted from ARS-30. The wording of the premise scene has been modified to measure the academic resilience level of pharmacy students. This scale removes 14 items and adds one dimension, with a Cronbach’s α coefficient for the total scale is 0.84 and values ranging from 0.61 to 0.82 for the subscales. And the Nursing Student Academic Resilience Scale by Ali-Abadi et al. (2021) is similar to the Pharmacy scale but specialized for nursing students, acknowledging the unique stressors in nursing education. This tool includes six dimensions and 24 items, demonstrating good content validity and aggregation validity. The Cronbach’s α coefficient for the entire scale is 0.88, with values of each dimension ranging from 0.63 to 0.78, indicating the reliability of the measurement tool.

Hoge et al. (2007) highlighted resilience as a multidimensional construct, best evaluated by an individual’s ability to recover from stressful experiences. The ARS-30, in particular, stands out for its specific context-based approach, enabling a more nuanced understanding of academic resilience in challenging academic environments. The ARS-30 has been revised in various versions to suit different contexts and populations, reflecting its versatility and applicability across diverse educational settings. For example, the United States version (Chisholm-Burns et al., 2019) was tailored for pharmacy students resulted in a Cronbach’s α coefficient of 0.84, indicating strong internal consistency. Additionally, the Turkish version (Ulas and Secer, 2020) applied in a high school setting, with a Cronbach’s *α* coefficient of 0.82, demonstrating its reliability in this demographic. Moreover, the Spanish version (Trigueros et al., 2020) targeted at nursing students, yielding a Cronbach’s *α* coefficient of 0.84, showing strong internal consistency. Each of these versions confirms the ARS-30’s robust internal consistency and validity across different contexts and student populations.

In 2023, the ARS-30 was specifically revised for Chinese high school students (Cui et al., 2023). This revision involved modifying the scenarios and retaining 18 of the original 30 items, while added one dimension and two new items to better align with the learning characteristics and needs of high school students. It was reported that the Cronbach’s *α* coefficient of the total scale is 0.90, with values for each dimension ranging between 0.73 and 0.83, indicating that the reliability and validity meet the requirements of psychometric tools. However, our study specifically focuses on academic resilience among Chinese students in higher education. Recognizing the differences in age, learning environments, and psychological conditions between high school and college students, it becomes essential to adapt the original ARS-30 accordingly. This adaptation aims to create a more fitting tool for assessing academic resilience in the Chinese higher education context. The scenario setting of the ARS-30 overcomes the limitations of using only item-level evaluations. Furthermore, through a series of processes such as localization and reliability and validity testing, the ARS-30 has the potential to become an effective and reliable assessment tool for evaluating the academic resilience of Chinese college students.

The objective of this study is to validate the Chinese version of the ARS-30 for students in Chinese higher education. It would empower educators and psychologists to more precisely measure the academic resilience of college students. This is crucial for the early detection of issues and enabling the provision of timely psychological interventions for students experiencing significant levels of stress.

To achieve this objective, the study proposes the following hypotheses: Firstly, it hypothesizes that the C-ARS-30 is suitable for assessing the level of academic resilience among Chinese university students. This validation will take into account the unique aspects of the Chinese higher education environment, evaluating the dimensions and items of ARS-30 to ensure that the scale accurately reflects the students’ resilience in the face of academic challenges. Secondly, given that academic resilience has been shown to correlate with academic self-efficacy and resilience (Cassidy, 2015; Tan et al., 2024), this study hypothesizes a positive correlation between academic resilience and both resilience and self-efficacy. This implies that students with higher academic resilience are likely to exhibit stronger levels of resilience and self-efficacy, aiding them in better coping with academic pressures. Through the validation of these hypotheses, the study aims to deepen the understanding of the concept of academic resilience and provide a theoretical basis for psychological interventions in higher education.

## 2 Methods

### 2.1 Settings and participants

An online survey was conducted using convenience sampling between January and March 2023 to obtain a sufficiently large sample size. For Exploratory Factor Analysis (EFA), the sample size of at least 300 is generally recommended (Worthington and Whittaker, 2006), while for Confirmatory Factor Analysis (CFA), a minimum sample size of 200 is required (Hair et al., 2013). Considering a potential invalid response rate of 20%, the minimum required sample size was set at 600. The participants were recruited from four higher education institutes (two undergraduate universities and two junior colleges) located in Guangzhou, Guangdong province. The inclusion criteria comprised full-time college students who had provided informed consent and voluntarily participated in the study. Exclusion criteria included individuals who had participated in the pilot test of the research or requested to withdraw from the study. A total of 1,650 questionnaires were collected, out of which 1,542 valid responded were obtained after eliminating incomplete information or highly similar responses, resulting in an effective response rate of 93.45%.

### 2.2 Measures

#### 2.2.1 Academic Resilience Scale-30, ARS-30

ARS-30, devised by Cassidy (2016), comprises three dimensions: perseverance (14 items), reflecting and adaptive-help-seeking (9 items), and negative influence and emotional response (7 items). A Likert 5-point scoring method was adopted, with a score of 1 indicating “strongly agree” and a score of 5 indicating “strongly disagree.” The total possible score ranges from 30 to 150, with higher scores representing greater levels of academic resilience. The correlation coefficient between ARS-30 and academic self-efficacy was 0.49, indicating good validity.

#### 2.2.2 10-item Connor Davidson Resilience Scale, CD-RISC-10

A streamlined iteration of the Connor-Davidson Resilience Scale (Connor and Davidson, 2003) developed by Campbell-Sills and Stein (2007), the CD-RISC-10 has been adapted for the Chinese context by Wang et al. (2010) scholar. This scale comprises a single dimension and 10 items. The Cronbach’s *α* coefficient for this scale in this study was 0.933.

#### 2.2.3 General Self-Efficacy Scale, GSES

The GSES, developed by Ralf and Mattias (1995), evaluates optimistic self-beliefs for coping with various challenging demands in life. GSES comprises 10 items that are rated on a four-point Likert scale, ranging from 1 (completely incorrect) to 4 (exactly right). Higher scores indicate a higher level of self-efficacy. The Chinese version of GSES was revised by Wang (2001), and psychometric results demonstrated its high reliability and validity. In this study, the Cronbach’s *α* coefficient for GSES was calculated to be 0.908.

### 2.3 Translation procedure

Prior to study commencement, approval was obtained from Cassidy of ARS-30 for its adaptation in a Chinese context. According to Brislin’s model of forward and backward translation (Jones et al., 2001), the ARS-30 was translated and adjusted. Initially, two bilingual translators who are native Chinese speakers with overseas study experience independently translated the ARS-30 from English to Chinese. In case there were discrepancies between the translations, the translators held a discussion online or face-to-face to arrive at a unified Chinese version. Subsequently, the above version was sent to two other experts who conducted research abroad and had never seen the original scale, producing two separate back-translated versions. Any differences were modified through a new round of translation-back translation until the final back version was achieved.

The expert committee consisted of six academic experts, each possessing a doctoral degree and more than 10 years of work experience in fields like statistics, psychology, sociology, or scale development. Experts were asked to evaluate the academic relevance and language consistency of each item using a 4-point scale that ranged from 1 (not at all relevant) to 4 (very relevant). After discussions between the researchers and the experts, some items were adjusted as follows: “I would just give up” was revised to “I would give up the tutors’ feedback” for improved clarity. “I would try to think more about my strengths and weaknesses to help me work better” was modified to “I would try to deeply think about my strengths and weaknesses to help me finish homework better” for greater precision. Incrementally, adjustments were made until the initial version of C-ARS-30 was completed, the scoring method remaining consistent with the original scale.

A pilot test was randomly conducted among 40 college students prior to the formal survey in order to assess the clarity of expressions, instructions, and scale items. Participants were asked about their perception of ambiguous words or phrases, as well as any responses they may have experienced with certain items, and completion time for the test was tested. The results showed that the initial version of C-ARS-30 was well-suited to Chinese expression patterns, and the average completion time for the scale was approximately 3 min.

### 2.4 Data collection

This study adopted a cross-sectional survey design. Initially, the researchers proactively contacted the school principals beforehand to apprise them of the study’s objectives and procedures. Afterwards, online questionnaire links (https://www.wjx.cn/) were provided to them, which they then passed on to their students for submission. Ethical approval was obtained from the Guangzhou Medical University Ethics Committee (No. L202212027). All participants received comprehensive information, including material on the purpose, participation method, and confidentiality principles of this study. Only individuals who voluntarily selected the option to “agree to participate” were authorized to complete the questionnaire. The completion time required was approximately 7–10 min. For the collection of retest reliability data, we randomly selected 100 college students from the total sample and sent them the questionnaire link via email after the initial assessment. Participants were provided with the same C-ARS-30 scale and were instructed to complete it under conditions similar to the initial test, responding within a specified timeframe. Out of the 100 students recruited, 89 completed the retest.

### 2.5 Data analysis

Statistical analysis was conducted using IBM SPSS 25.0 and Mplus 8.1. Descriptive statistics were used to analyze the demographic information. Validity assessments included content validity, construct validity, convergent and discriminant validity, as well as criterion-related validity. The construct validity of the scale was evaluated through both CFA and EFA. Further, Exploratory Structural Equation Modeling (ESEM) was applied to intensify the examination of the C-ARS-30’s construct validity. This involved the use of maximum likelihood estimation (ML) and Geomin rotation in the analytical model. The sample set was randomly divided into three segments (Sample I, Sample II, and Sample III) using SPSS, specifically for the EFA, CFA, and ESEM analyses, respectively. The efficacy of CFA and ESEM models was evaluated based on several indices, including relative chi-square (*X^2^/df* ≤ 5), comparative fit index (CFI ≥ 0.90), Tucker–Lewis index (TLI ≥ 0.90), root mean square error of approximation (RMSEA ≤ 0.08) and standardized root mean square residual (SRMR ≤ 0.08) (Bentler and Bonett, 1980; Hu and Bentler, 1999; Schumacker and Richard, 2004). To assess the internal reliability and stability of the scale, Cronbach’s alpha coefficients and test–retest reliability methods were applied. In line with established standards, reliability values exceeding 0.70 were acceptable. For all statistical tests *p* < 0.05 was considered significant.

## 3 Results

### 3.1 Participants characteristics

The study sample consisted of 504 males (32.7%) and 1,038 females (67.3%), aged ranged from 18 and 24 years, with a mean age of 19.02 ± 0.96 years. The participants were categorized into different academic years, including 586 first-year college students (freshmen), 554 s-year college students (sophomores), 290 third-year college students (juniors), and 112 fourth-year college students (seniors). Additionally, 1,051 participants (68.2%) were from medical specialties (see Table 1).

**Table 1:** Demographic characteristics of college students (*n* = 1,542).

Variables	*n*	%
Gender		
Male	504	32.7
Female	1,038	67.3
School type		
University	895	58.0
Technical or vocational colleges	647	42.0
Profession		
Medical specialty	1,051	68.2
Non-medical specialty	491	31.8
Grade		
First-year	586	38.0
Second-year	554	35.9
Third-year	290	18.8
Fourth-year	112	7.3
Resident		
City	902	58.5
Rural	640	41.5

### 3.2 Validity analysis

#### 3.2.1 Content validity

Content validity was evaluated using two indices: the Item-Content Validity Index (I-CVI) and the Scale-Content Validity Index (S-CVI). Researchers recommend an I-CVI of 0.78 or higher when evaluated by six or more experts, and an S-CVI/Ave of 0.90 or higher on the scale indicate excellent content validity (Lynn, 1986). The results showed that the CVI value of each item ranged from 0.833 to 1.000, and the CVI value of the total scale (S-CVI/Ave) was 0.986, suggesting an acceptable level of content validity.

#### 3.2.2 CFA

**Figure 1:**
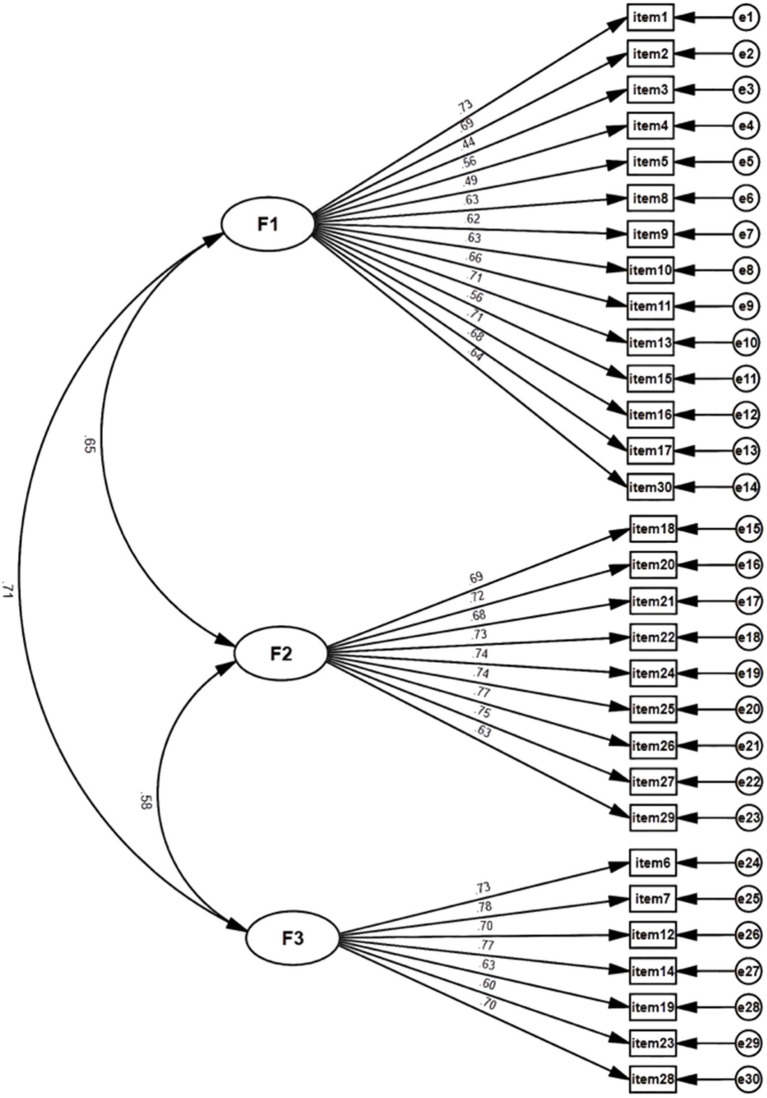
Confirmatory factor analysis of the structure of original ARS-30. (*N* = 514).

The CFA was conducted on Sample I, adhering to the structure of the original scale and utilizing the maximum likelihood estimation method. However, the results indicated insufficient model fit indices (*χ*^2^ = 2702.523; SRMR = 0.088; RMSEA = 0.107; CFI = 0.742; TLI = 0.714). Additionally, the factor loading ranges from 0.44 to 0.78, as illustrated in Figure 1. These findings suggest that the model currently lacks a satisfactory fit to the data, and the factor loadings indicate limited associations between the items and their respective factors.

#### 3.2.3 EFA

**Figure 2:**
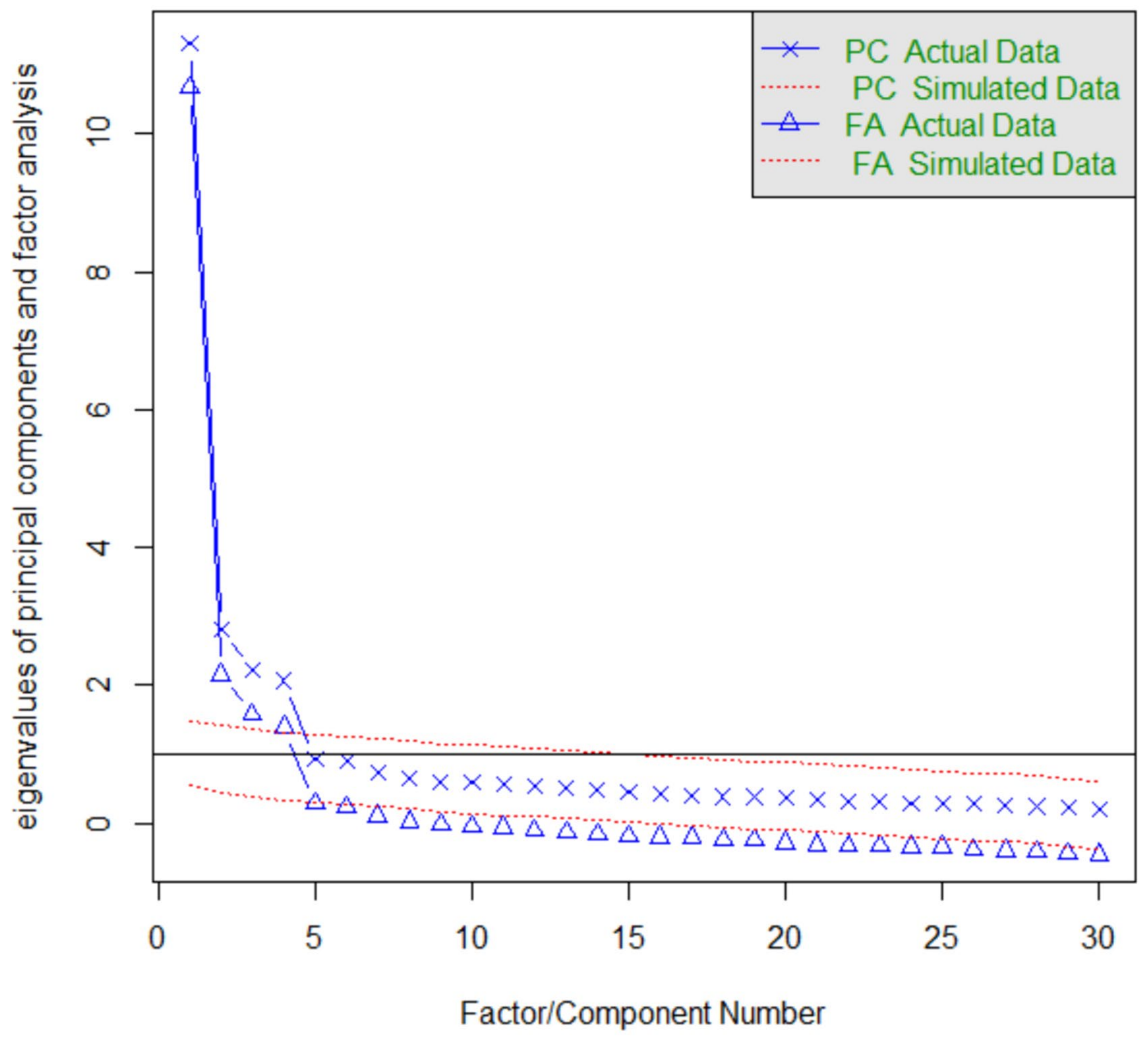
Parallel analysis scree plot.

To explore the structure of C-ARS-30, parallel analysis was conducted on Sample I to explore the number of factors. In the lithotripsy diagram, the solid line denotes actual data, while the dashed line signifies simulated data. Principal Component Analysis reveals that the four components in the actual data exhibit higher values than those in the simulated data. Similarly, in Factor Analysis, depicted by the triangular line, four factors within the actual data exhibit eigenvalues that exceed the mean eigenvalue derived from 100 simulated data matrices (refer to Figure 2). This analysis led to the logical decision to divide the C-ARS-30 into four distinct dimensions.

Following this, EFA was conducted using SPSS software. The results of EFA showed that KMO was 0.940, Bartlett’s test reached a significant level (*χ^2^* = 9175.859, *p* < 0.001), and four common factors were obtained, with a cumulative variance contribution rate of 61.320%, indicating that the factor analysis was justified in the sample. The EFA results indicated that the corresponding items for the four factors are as follows: Factor 1 includes items 18, 20, 21, 22, 23, 24, 25, 26, 27, and 29; Factor 2 includes items 5, 6, 7, 12, 14, 15, 19, and 28; Factor 3 includes items 1, 2, 11, 13, 16, 17, and 30; Factor 4 includes items 3, 4, 8, 9, and 10. Based on the contents of each common factor and with reference to the original scale, four factors were labeled as “self-reflection and help-seeking,” “negative influence and emotional response,” “perseverance,” and “adaptive thought processes.” Compared to the original ARS-30, entry 23, “I would stop myself from panicking,” has been reassigned from the “negative influence and emotional response” dimension to the “self-reflection and help-seeking” dimension. Additionally, entries 3, 4, 8, 9, and 10 have been reclassified to form a new dimension called “adaptive thought processes,” replacing the original “perseverance” dimension.

#### 3.2.4 CFA and ESEM analysis

**Figure 3:**
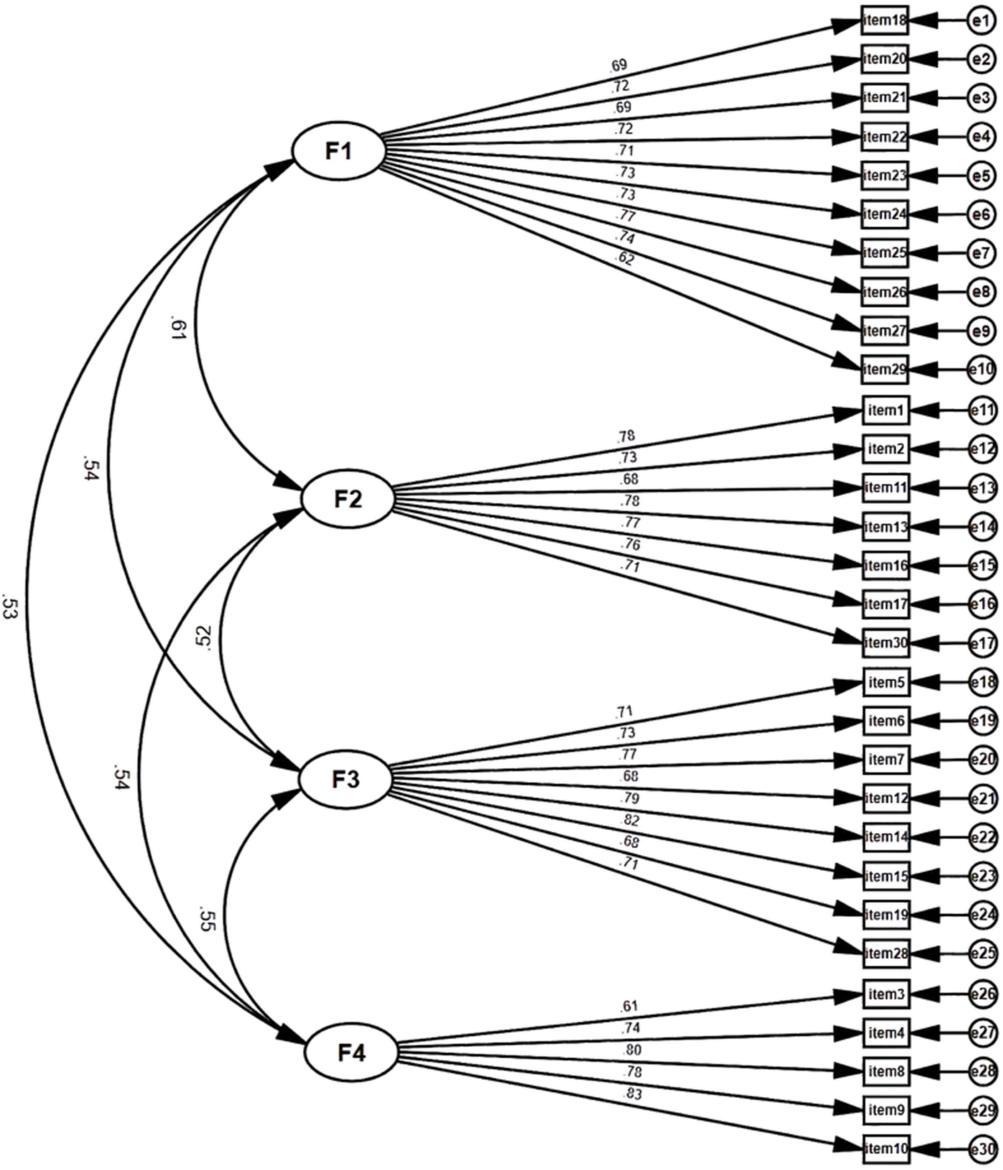
Confirmatory factor analysis of the C-ARS-30. (*N* = 514).

To further validate the results of the identified structures from EFA, CFA was conducted on Sample II, and ESEM analysis was performed on Sample III. The CFA factor loading ranges from 0.61 to 0.83, as illustrated in Figure 3. The factor loadings resulting from both CFA and ESEM for C-ARS-30 are presented in Table 2. Notably, the fit indices of the ESEM model are superior to those of the CFA model, meeting established standards (*χ^2^/df* = 2.647, CFI = 0.937, TLI = 0.915, RMSEA = 0.057, SRMR = 0.027) (see Table 3).

**Table 2:** CFA and ESEM factor loadings of C-ARS-30 (*N* = 514).

	CFA					ESEM			
	Loading	SE	Z(CR)	AVE	CR	F1	F2	F3	F4
ITEM18	0.687	0.025	26.956	0.509	0.912	0.099	0.052	0.620	0.079
ITEM20	0.720	0.023	30.749			0.063	0.017	0.649	0.036
ITEM21	0.688	0.025	27.004			0.148	0.158	0.578	−0.139
ITEM22	0.724	0.023	31.153			0.089	0.028	0.649	0.095
ITEM23	0.711	0.024	29.479			0.131	0.129	0.627	0.033
ITEM24	0.726	0.023	31.279			−0.141	0.034	0.810	0.052
ITEM25	0.730	0.023	31.845			−0.013	−0.145	0.780	0.023
ITEM26	0.772	0.020	38.107			0.155	0.034	0.638	−0.037
ITEM27	0.743	0.022	33.533			−0.118	−0.047	0.804	0.012
ITEM29	0.621	0.029	21.257			−0.036	−0.149	0.724	−0.028
ITEM1	0.777	0.020	38.024	0.555	0.897	0.720	0.170	−0.198	0.094
ITEM2	0.731	0.023	31.263			0.804	−0.100	−0.083	0.123
ITEM11	0.680	0.026	25.820			0.627	−0.024	0.112	0.029
ITEM13	0.780	0.020	38.708			0.666	0.029	0.186	−0.074
ITEM16	0.772	0.021	37.477			0.680	0.045	0.119	−0.010
ITEM17	0.763	0.021	35.831			0.720	0.081	0.077	−0.145
ITEM30	0.708	0.025	28.831			0.679	−0.046	0.070	0.012
ITEM5	0.706	0.025	28.769	0.539	0.903	−0.088	0.625	−0.001	0.281
ITEM6	0.726	0.024	30.435			−0.014	0.619	−0.033	0.298
ITEM7	0.770	0.021	36.507			−0.013	0.637	−0.012	0.366
ITEM12	0.676	0.026	25.630			0.093	0.610	0.090	−0.097
ITEM14	0.785	0.020	40.173			0.003	0.767	0.049	−0.024
ITEM15	0.815	0.018	45.850			0.027	0.745	0.020	0.075
ITEM19	0.675	0.026	25.480			0.050	0.696	0.011	−0.024
ITEM28	0.707	0.025	28.810			0.076	0.604	0.101	0.002
ITEM3	0.612	0.031	19.695	0.573	0.869	0.018	0.037	−0.019	0.650
ITEM4	0.744	0.023	32.016			0.253	−0.057	0.005	0.645
ITEM8	0.800	0.020	40.026			0.296	−0.032	0.026	0.515
ITEM9	0.777	0.022	36.009			−0.011	0.075	0.144	0.735
ITEM10	0.832	0.018	46.788			0.161	0.061	0.139	0.607

F1 = self-reflection and help-seeking; F2 = negative influence and emotional response; F3 = perseverance; F4 = adaptive thought processes.

**Table 3:** Model fit indices of C-ARS-30.

	*χ^2^*	*df*	SRMR	RMSEA (95% CI)	CFI	TLI
CFA	1437.073	399	0.056	0.071(0.067,0.075)	0.884	0.874
ESEM	849.614	321	0.027	0.057(0.052,0.061)	0.937	0.915

#### 3.2.5 Convergent and discriminant validity

The CFA results, as presented in Table 2, offer a detailed validation of the C-ARS-30’s convergent validity. The Average Variance Extracted (AVE) values for the four identified factors (0.509, 0.555, 0.539, and 0.573) notably exceed the minimum threshold of 0.5. The Construct Reliability (CR) values, which are 0.912, 0.897, 0.903, and 0.869, respectively, all well above the acceptable standard of 0.7, indicating a high level of internal consistency within each factor. Additionally, the factor loadings for each item exceed 0.6, denoting robust correlations between the items and their respective factors, and thereby establishing a strong level of convergent validity for the scale. Table 4 reveals that the square root of the AVE values for each dimension exceeds the Pearson correlation values for that dimension and other dimensions (refer to Table 4). This finding suggests that the discriminant validity meets the established standard, and the differences between different dimensions are within a reasonable range. In other words, the factors exhibit sufficient distinctiveness, indicating that the C-ARS-30 effectively measures different aspects of academic resilience.

**Table 4:** Discriminant validity analysis of the scale.

	F1	F2	F3	F4
F1	**0.713**			
F2	0.557**	**0.745**		
F3	0.487**	0.475**	**0.734**	
F4	0.460**	0.471**	0.483**	**0.757**

**p < 0.01. F1, F2, F3, and F4 denote factor 1, factor 2, factor 3, and factor 4 correspondingly. The diagonal represents the square root of AVE value.

#### 3.2.6 Criterion-related validity

The CD-RISC-10 and GSES were used as criterion-validity measures. The findings revealed a significant positive association between C-ARS-30, CD-RISC-10 and GSES (*r* = 0.542, 0.488, *p* < 0.01).

### 3.3 Reliability analysis

#### 3.3.1 Internal consistency

The total Cronbach’s *α* coefficient of the C-ARS-30 was 0.930, and the Cronbach’s *α* coefficient of each dimension ranged from 0.780 to 0.900. These high coefficients indicate strong internal consistency within the scale, ensuring that the items in each dimension are closely related and measure the same construct. The split-half reliability of the scale was 0.810 and the split-half coefficients of each dimension ranged from 0.754 to 0.884. These results also reflect satisfactory reliability, demonstrating that the scale is consistent and reliable.

#### 3.3.2 Test–retest reliability

The researchers used online email to randomly recruit 100 college students for a retest 3 weeks after the initial assessment. Out of the 100 students, 89 completed the retest. The results showed that the retest reliability coefficient was 0.794 (*p* < 0.05), within the acceptable range. This indicates that the scale’s scores remain stable over time and are reliable for repeated measurements.

## 4 Discussion

Academic resilience applies traditional concepts of resilience to academic settings. It refers to the ability of individuals to achieve academic success and demonstrate high levels of performance, particularly when facing challenging life circumstances that might otherwise lead to academic failure and dropout (Rudd et al., 2021). To date, an authoritative and readily available assessment tool for academic resilience amongst Chinese students in higher education has not been recognized, with existing tools merely addressing certain aspects, without focus on the experience of stress (Li et al., 2017). Despite several recent studies on academic resilience in China, there remains a dearth of comprehensive tools capable of assessing the intricate dimensions of college students’ academic resilience. Therefore, this study diligently followed the standardized process of questionnaire localization to a Chinese iteration of ARS-30. Our findings substantiate that the C-ARS-30 is an effective and dependable instrument for gauging students’ academic resilience levels in higher education. Early detection of lower levels of academic resilience may help facilitate timely interventions and support to bolster academic development and success.

### 4.1 Validity analysis

The C-ARS-30, in this study, demonstrates robust validity, particularly in the context of academic resilience, a complex, multi-dimensional construct. The scale’s association with other psychological assessment instruments has been extensively examined, underscoring its relevance in academic settings. For instance, previous research by Cassidy (2016) identified a moderate parallel validity (*r* = 0.49) between academic resilience and academic self-efficacy in college students. However, Grande’s investigation into the correlation between academic resilience and quality of life did not yield a significant relationship (Grande et al., 2022). This study reveals a significant positive correlation between C-ARS-30 scores and both resilience and self-efficacy among college students (*r* = 0.542, 0.488). This indicates that higher levels of academic resilience correlate with stronger resilience and a heightened sense of self-efficacy, supporting previous findings by Martin and Marsh (2006) and Cassidy (2015). Given the myriad academic challenges faced by college students, which can lead to psychological distress and affect learning motivation (Thorsen et al., 2021), fostering academic resilience becomes essential. This not only aids in maintaining students’ mental health but also enhances their motivation and academic performance.

This study adopts a meticulous research methodology, initiating with the structure of the original ARS-30, and progresses to explore and validate the factor structure of its Chinese version, C-ARS-30, within the nuances of Chinese cultural context. The structural validity of the C-ARS-30 was rigorously evaluated using both CFA and ESEM analyses.

The CFA, a traditional method for validating theoretical factor structures as proposed by Jöreskog (1969), initially presented suboptimal fit indices (CFI = 0.884, TLI = 0.874) for the C-ARS-30. This method predefines a model structure and attempts to confirm it with empirical data, potentially leading to poor fit if the model is overly complex or misaligned with the actual data. CFA typically assumes a simplistic, idealized structure with “pure factors,” where each variable is exclusively associated with a single predetermined factor, without considering possible cross-loadings (Asparouhov and Muthén, 2009). In contrast, ESEM, which integrates the strengths of both CFA and EFA, provided a more accommodating approach. This method offers greater flexibility, making it adept at handling complex datasets (van Zyl and Ten Klooster, 2022). Particularly useful in cross-cultural research, ESEM can effectively address variations in item phrasing and interpretation across different cultural groups (van Zyl et al., 2022).

The divergent results between the CFA and ESEM in this study likely stem from the rigid constraints of CFA’s model configuration and the distribution of the sample. The findings indicate that the ESEM’s adaptable nature more aptly captures the multifaceted and intricate nature of the C-ARS-30, aligning more closely with the actual structure of the data and robustly supporting the scale’s multi-dimensional division. Ultimately, the validated C-ARS-30 encompasses four distinct dimensions and 30 items. Significant modifications have been made to both the scale’s dimensions and items in this adapted version, setting it apart from the original ARS-30. These adjustments ensure that the C-ARS-30 accurately reflects the specific contexts and cultural considerations relevant to Chinese higher education students, providing a reliable tool for assessing academic resilience in this population.

The dimension of self-reflection and help-seeking includes 10 entries, such as “I would use my past successes to help motivate myself” and “I would seek help from my tutors.” This dimension reflects the characteristics of students who engage in self-reflection and seek assistance from others when facing significant challenges. This behavior is similar to the resilience characteristics reported by Lamond et al. (2009) regarding adaptability. Notably, entry 23, “I would stop myself from panicking, “was included in this dimension, adopted from the original dimension of negative influence and emotional response. According to Richardson’s (2002) resilience process theory, college students experience a state of bio-psychological-spiritual homeostasis, known as the comfort zone when not facing academic challenges. However, exposure to stressors or times of adversity, like the COVID-19 global pandemic, can disrupt this equilibrium, triggering physical and mental reactions. Maladjusted students are particularly vulnerable to emotional distress, such as anxiety and feelings of panic. Besides addressing problem-solving, individuals also need to consider coping strategies for adverse emotions. Individuals with higher levels of resilience can mobilize multiple protective factors to withstand life’s stimuli, engage in self-integration, overcome difficulties, and restore biopsychospiritual homeostasis. The wording of this item 23 aligns with the principles of reflection and help-seeking, rather than a negative emotional reaction.

In the refinement of the C-ARS-30, particularly within the dimension of perseverance, entries 3, 4, 8, 9, and 10 have been restructured into a new subscale named “adaptive thought processes.” According to the seminal Cognitive Phenomenological Transactional (CPT) model (Farrington, 1995), individuals facing external environmental threats or stimuli evaluate stressors based on their mindset and assess the interplay between people, events, environment, and other factors in stressful situations to determine coping behaviors. The items in this dimension, such as “I would see the situation as temporary” and “I would see the situation as a challenge,” indicate adaptive thinking and problem-centered stress coping mechanisms (Folkman and Moskowitz, 2004), rather than mere perseverance. This perspective is akin to the modified version of the ARS-30 by Chisholm-Burns et al. (2019). Specifically, items 3, 4, and 9 are categorized under the ‘adaptive thought processes’ dimension, reflecting their role in eliciting responses to academic challenges. Item 3, “I would just give up,” suggests a passive response to difficult. While this may initially seem non-adaptive, it acknowledges personal limits within this framework. Item 4, “I would use the situation to motivate myself,” on the other hand, denotes a positive approach to challenges, using adversity as a source of motivation, which resonates with Keller’s (1983) ARCS model of motivation. Item 9, “I would do my best to stop thinking negative thoughts,” advocates for a proactive stance in managing and redirecting negative thoughts associated with challenges, thereby promoting a positive mindset.

This “adaptive thought processes” dimension, therefore, encapsulates a spectrum of responses to challenges, emphasizing the cognitive and emotional strategies employed by individuals in academic contexts. It highlights the importance of flexibility in thinking and the ability to adjust strategies in diverse situations, leading to successful outcomes. Usher Am et al.’s (2023) research further supports this notion, illustrating how nursing students during the COVID-19 pandemic adapted to significant disruptions in their education by employing problem-solving and emotion regulation strategies. This adaptability is a hallmark of academic resilience, enabling students to navigate and overcome diverse challenges.

Lastly, the dimension of negative influence and emotional response largely aligns with the original scale’s emotional subscale, incorporating two new entries. The items “I would change my career plans” and “I would blame the tutor” were added to acknowledge the varied interpretations and reactions to academic frustration among students with different social and educational backgrounds. These additions reflect the complex nature of academic resilience and the range of responses it may evoke in students facing educational challenges.

### 4.2 Reliability analysis

The C-ARS-30 exhibited commendable reliability in this study, as indicated by its performance across various reliability metrics. Reliability assessment traditionally hinges on three critical aspects: stability, internal consistency, and homogeneity.

In terms of internal consistency, both the Cronbach’s *α* coefficient and the split-half reliability coefficient for the C-ARS-30 were above the 0.70 threshold. This surpasses the minimum standard often cited for reliable measurement instruments, thereby confirming the scale’s strong internal consistency. Such a level of internal consistency is vital as it implies that the scale items are coherently measuring the same underlying construct without significant deviation.

Nevertheless, the aspect of stability, as reflected in the retest reliability score of 0.775 over a three-week interval, suggests room for improvement. Stability, a measure of the consistency of results over time, is crucial for ensuring that a scale can reliably measure a construct across different time points. The moderate retest reliability score points to potential variability in the scale’s measurements over time. It’s important to acknowledge that academic resilience, akin to constructs like attitude and emotion, is categorized as an unstable latent variable within psychology. This means that it is subject to fluctuations due to various external influences such as environmental factors or intervention measures. Consequently, the observed variability in the retest reliability could be attributed to the inherent nature of academic resilience as a construct that can evolve or be influenced by external factors.

Therefore, future research should delve deeper into examining the stability of the C-ARS-30 over longer periods and under varying conditions. This would involve assessing how different intervention strategies or changes in the educational and personal environment of students might impact their levels of academic resilience as measured by the scale. Such investigations are crucial for understanding the dynamic nature of academic resilience and for refining the scale to enhance its reliability and applicability in diverse educational settings.

## 5 Limitations and future research

This study’s limitations, which merit acknowledgment, have implications for its interpretation and application.

Firstly, the adoption of convenience sampling method and the focus on a specific subset of students from limited geographical areas could potentially introduce sampling bias. Moreover, the use of a non-probabilistic sampling method makes it difficult to ensure gender balance in the sample. This bias may affect the applicability of our findings to a broader population. It is recommended that subsequent studies include more diverse and representative samples. This approach should involve students from a variety of regions and educational backgrounds across China, ensuring a more comprehensive understanding of academic resilience within the broader Chinese higher education context. Additionally, future investigations should use gender measurement invariance tests to ascertain whether the C-ARS-30 functions similarly across male and female subjects.

Secondly, while the C-ARS-30 exhibited strong psychometric properties and structural validity in this research, it is important to consider that the scale’s factor structure may exhibit variations across different student demographics or educational settings. Therefore, future research should focus on examining the stability and consistency of the C-ARS-30’s factor structure in varied populations and contexts. This would contribute to verifying the scale’s construct robustness and its adaptability to different educational environments and student groups.

Thirdly, there is a valuable opportunity to delve deeper into the influence of demographic and social factors on academic resilience in higher education students. An exploration of variables such as age, gender, academic discipline, socio-economic status, and previous educational experiences could provide deeper insights into the dynamics of academic resilience. Understanding how these factors might moderate or mediate the manifestation of academic resilience can offer a richer, more nuanced understanding of the construct. This could, in turn, inform targeted interventions and support strategies tailored to specific student needs and contexts, thereby enhancing the effectiveness of educational and psychological support services in higher education institutions.

## 6 Conclusion

The utilization of the Chinese version of the ARS-30 presents a significant opportunity for educators in China to accurately assess academic resilience among students. This assessment is crucial for identifying students who may exhibit lower levels of resilience, thereby facilitating the implementation of targeted interventions designed to bolster their academic progression. We advocate for the integration of academic resilience assessment into the educational framework, which would serve as a vital tool in monitoring and enhancing students’ resilience levels.

The C-ARS-30 has demonstrated exceptional reliability and validity in this study. Each subscale measures a distinct aspect of academic resilience, providing a comprehensive assessment of students’ ability to cope with academic challenges. Given the prevalent high stress levels among Chinese students, the C-ARS-30 is poised to function as a crucial diagnostic tool. It can identify students’ non-adaptive responses to academic stressors and lay the groundwork for developing customized, resilience-enhancing interventions. By proactively focusing on academic resilience, educators can significantly contribute to improving students’ coping mechanisms and overall well-being, thereby fostering academic success and personal development.

Furthermore, the C-ARS-30 offers a promising direction for future research in academic resilience. It provides a valuable asset for educational institutions in China, aiding them in effectively supporting their students throughout their academic journey. By nurturing academic resilience, educators can equip students with essential skills to navigate challenges, adapt effectively to various stressors, and flourish in their academic endeavors. This focus on resilience building is not only pivotal for academic success but also for fostering well-rounded, resilient individuals capable of handling the complexities of the modern educational landscape.

## Data availability statement

The raw data supporting the conclusions of this article will be made available by the authors, without undue reservation.

## Ethics statement

The studies involving humans were approved by Guangzhou Medical University Ethics Committee (No. L202212027). The studies were conducted in accordance with the local legislation and institutional requirements. Written informed consent for participation in this study was obtained from the participants.

## Author contributions

W-yT: Writing – original draft, Conceptualization. J-nC: Writing – review & editing. S-hL: Data curation, Investigation, Writing – original draft. C-qL: Writing – original draft, Formal analysis. QL: Formal analysis, Writing – original draft. YM: Writing – original draft, Investigation. YZ: Writing – original draft, Funding acquisition, Project administration, Supervision. TW: Investigation, Methodology, Writing – original draft. H-fC: Investigation, Methodology, Writing – original draft. L-qS: Investigation, Writing – original draft. C-yM: Investigation, Writing – original draft. J-wC: Data curation, Writing – original draft. GS: Writing – review & editing.
